# EVALI – E-Cigarette or Vaping Product Use-Associated Lung Injury: A Case Report

**DOI:** 10.7759/cureus.13541

**Published:** 2021-02-24

**Authors:** Ramesh Adhikari, Thoyaja Koritala, Raghavender Gotur, Srikrishna V Malayala, Nitesh K Jain

**Affiliations:** 1 Hospital Medicine, Franciscan Health, Lafayette, USA; 2 Geriatrics, Brown University, Providence, USA; 3 Internal Medicine, Mayo Clinic, Mankato, USA; 4 Internal Medicine, Cape Fear Valley Harnett Hospitalist Group, Lillington, USA; 5 Internal Medicine, Temple University Hospital, Philadelphia, USA; 6 Critical Care Medicine, Mayo Clinic, Mankato, USA

**Keywords:** e-cigarette and vaping product use associated lung injury (evali), electronic cigarettes' e-cigarettes' vaping' e-smoking, vitamin e, cdc evali, lung infiltrates, tetrahydrocannabinol, e-cigarette smoking, cannabinoids, public health, acute hypoxic respiratory failure

## Abstract

The use of electronic cigarettes among the young adult and adolescent population has increased over the past decade. Vaping is the process of inhaling an aerosol that is produced by heating a liquid or wax containing substances, such as nicotine, cannabinoids (e.g., tetrahydrocannabinol (THC), cannabidiol), flavoring, and additives (e.g., glycerol, propylene glycol) using an e-cigarette. A multistate epidemic associated with vaping prompted the Centers for Disease Control and Prevention (CDC) to issue an official health advisory on e-cigarette or vaping product use-associated lung injury (EVALI). EVALI is a diagnosis of exclusion with no specific diagnostic test.

We present a case of EVALI before the COVID-19 pandemic time in a 23-year-old immunocompetent male student with an eight-year history of vaping. He presented to the emergency department with fever, shortness of breath, tachypnea, nausea, and diarrhea. The patient had no past medical history. The patient denied illicit drug abuse or known drug allergies. The patient was admitted with a diagnosis of sepsis and pneumonia. The patient’s urine drug screen was positive for cannabinoids with a history of vaping. Community-acquired pneumonia due to Legionella, Pneumococcal, Mycoplasma bacteria was ruled out. Influenza A/B, Parainfluenza, Rhino, and Adenoviruses were negative. A computed tomographyscan of the chest showed bilateral infiltrates. He was treated with high dose steroids, empiric antibiotics, high flow oxygen and managed in ICU for seven days. The patient was discharged on tapering doses of steroid and counseled to quit vaping.

EVALI outbreak is strongly linked to vitamin E acetate in vaping products. EVALI is a diagnosis of exclusion with a history of vaping and responds well to steroids.

## Introduction

E-cigarette or vaping product use-associated lung injury (EVALI) is a diagnosis of exclusion with no specific diagnostic test carrying significant morbidity and mortality [1]. A total of 2,807 hospitalized EVALI cases or deaths are reported to the Centers for Disease Control and Prevention (CDC) from all 50 states, the District of Columbia, and two U.S. territories (Puerto Rico and U.S. Virgin Islands) as of February 18, 2020. Among 29 states and the District of Columbia, 68 deaths have been confirmed by CDC as of February 18, 2020 [2].

A sharp increase in emergency department (ED) visits related to e-cigarette or vaping products during August 2019 and peaking in September continue to decline after [2]. The decline in ED visits is likely multifactorial - increased public awareness of the risk associated with tetrahydrocannabinol (THC) containing e-cigarette or vaping product use due to rapid public health response, removal of vitamin E acetate from some products, and law enforcement actions related to illicit products. CDC stopped collecting data from states for EVALI cases as of February 2020 due to a decline in EVALI cases and deaths and due to identification of the primary cause of EVALI [2].

Based on state and national data from patient reports and product sampling, THC containing e-cigarette or vaping products, particularly from informal sources like friends, family, or in-person or online dealers, are linked to most EVALI cases. Vitamin E acetate was found in product samples tested by FDA and state laboratories from geographically diverse states and was also present in patient fluid samples tested by CDC [2].

Vitamin E acetate is strongly linked to EVALI outbreak [2]. Other chemicals of concern, including chemicals in either THC or non-THC product contribution, could not be ruled out due to lack of sufficient evidence [2]. Bronchoalveolar lavage (BAL) fluid samples (fluid samples collected from the lungs) were analyzed from 51 EVALI cases from 16 states and a comparison group from 99 individuals without EVALI for vitamin E acetate, plant oils, medium-chain triglyceride (MCT) oil, coconut oil, petroleum distillates, and diluent terpenes [2,3]. Vitamin E acetate was identified from 48 of the 51 EVALI patients but not in the BAL fluid from the healthy comparison group. No other toxicants were found in BAL fluid from either group, except for coconut oil and limonene (one EVALI patient each) [2,3].

## Case presentation

A 23-year-old immunocompetent male patient with an eight-year history of vaping presents to the emergency room with a three-day history of fever, shortness of breath, tachypnea, nausea, and diarrhea. The patient denies any past medical or surgical history. The patient denies any known drug or seasonal allergies. The patient denies any family history of lung diseases or asthma. The patient is a college student and denies using any prescription or over-the-counter medications. On his initial presentation, the patient was hypoxic and septic.

His vitals at the time of presentation were: Maximum temperature - 102.7 Fahrenheit, pulse rate - 119 beats per minute, blood pressure - 124/82 mmHg, respiratory rate - 24-28 breaths per minute.

Laboratory values

Arterial blood gas on 6 liters of nasal canal oxygen was: pH - 7.47 (7.35-7.45), CO2 - 33 (35-45 mmHg), oxygen - 68 (80-100 mmHg), bicarbonate - 24 (22-26 mEq/L).

Basic metabolic panel - sodium, potassium, chloride, bicarbonate, calcium levels were within normal limits. Serum creatinine was elevated. Liver function tests (LFT) were within normal limits. Complete blood picture with differential showed elevated white count with neutrophil predominance. Eosinophilia was absent. Erythrocyte sedimentation rate (ESR), C-reactive protein (CRP), procalcitonin were elevated. Serum lactic acid was normal. Viral panel - Influenza A/B, Parainfluenza, Rhino, and Adenovirus - was negative. Urine Legionella antigen, Pneumococcal Antigen, Mycoplasma IgM antibody were negative. Blood cultures (two sets) were negative. Urine toxicology was positive for cannabinoids (Table 1).

**Table 1:** Pertinent patient laboratory values.

Parameters	Normal Values	Patient's lab values
WBC	4.5-12 x 10^3^/uL	14.8 x 10^3^/uL
Neutrophils	43-82%	92%
Lymphocytes	15-45%	1%
Hemoglobin	13-17.7 g/dL	13.2 g/dL
			Platelets	150-350 x 10^3^/uL	276 x 10^3^/uL
Creatinine	0.60-1.20 mg/dL	1.45 mg/dL			
			Glomerular filtration rate	>60 ml/min/1.73 m^2^	60 ml/min/1.73 m^2^
Blood glucose	80-100 mg/dL	108 mg/dL			
			Lactate	0.5-2.0 mmol/L	1 mmol/L
Procalcitonin	<0.05 ng/mL	2.13 ng/mL			
			Erythrocyte sedimentation rate	0-15 mm/hr	97 mm/hr
C-reactive protein	<10 mg/L	35 mg/dL			
B-natriuretic peptide (BNP)	0.0-100 pg/mL	37 pg/mL

Imaging

**Figure 1:**
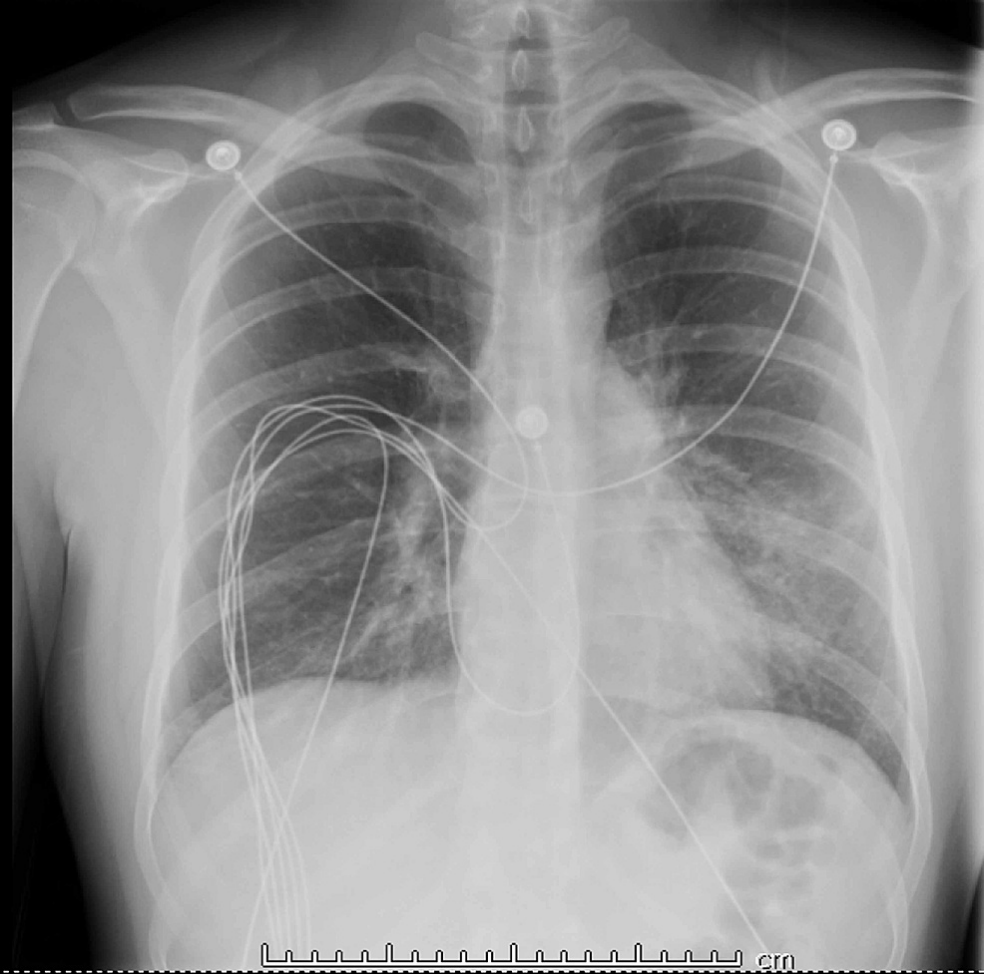
Chest X-ray - Bilateral lung infiltrates

**Figure 2:**
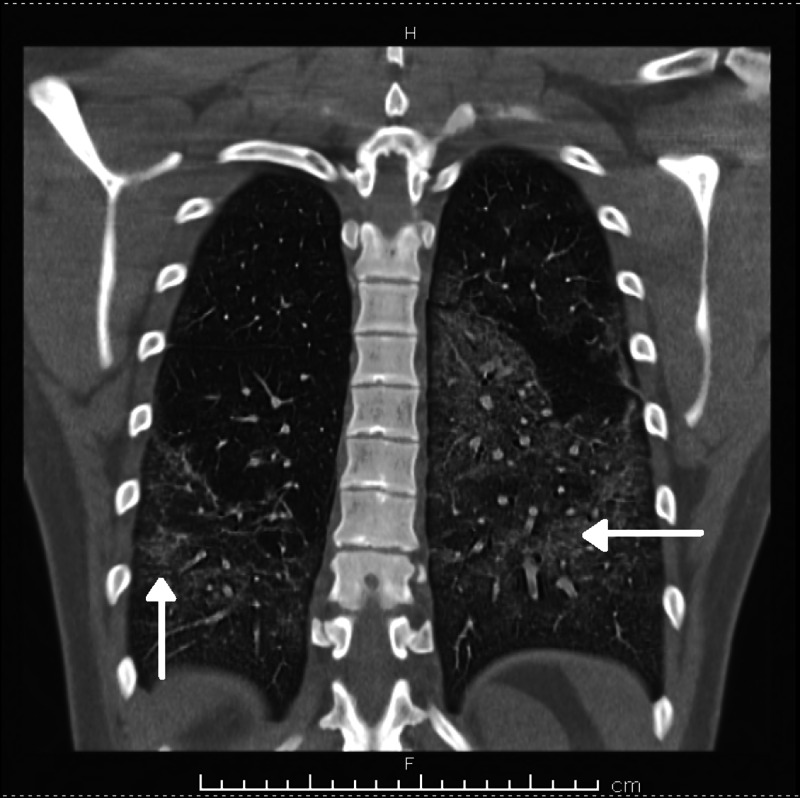
Computed tomography (CT) chest (coronal view) - Bilateral lung infiltrates due to EVALI EVALI: E-cigarette or vaping product use-associated lung injury

**Figure 3:**
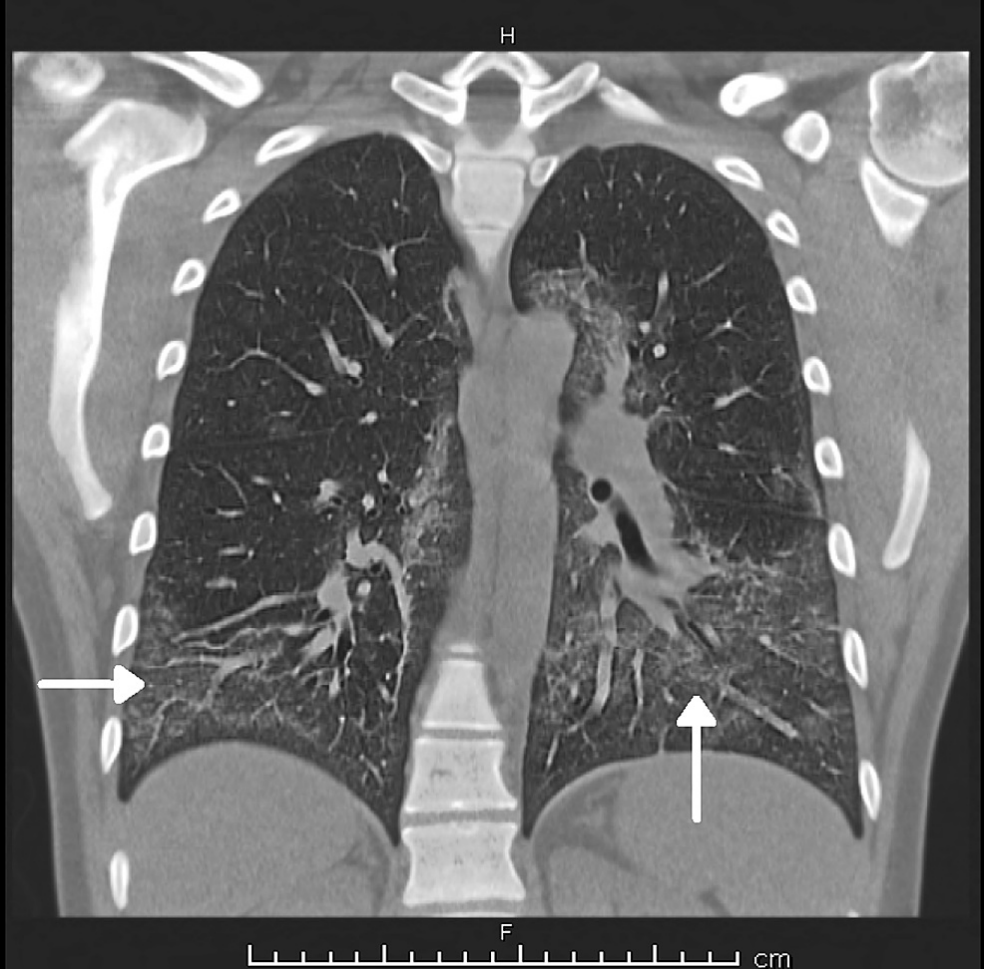
Computed tomography (CT) chest (coronal view) - Bilateral lung infiltrates due to EVALI EVALI: E-cigarette or vaping product use-associated lung injury

**Figure 4:**
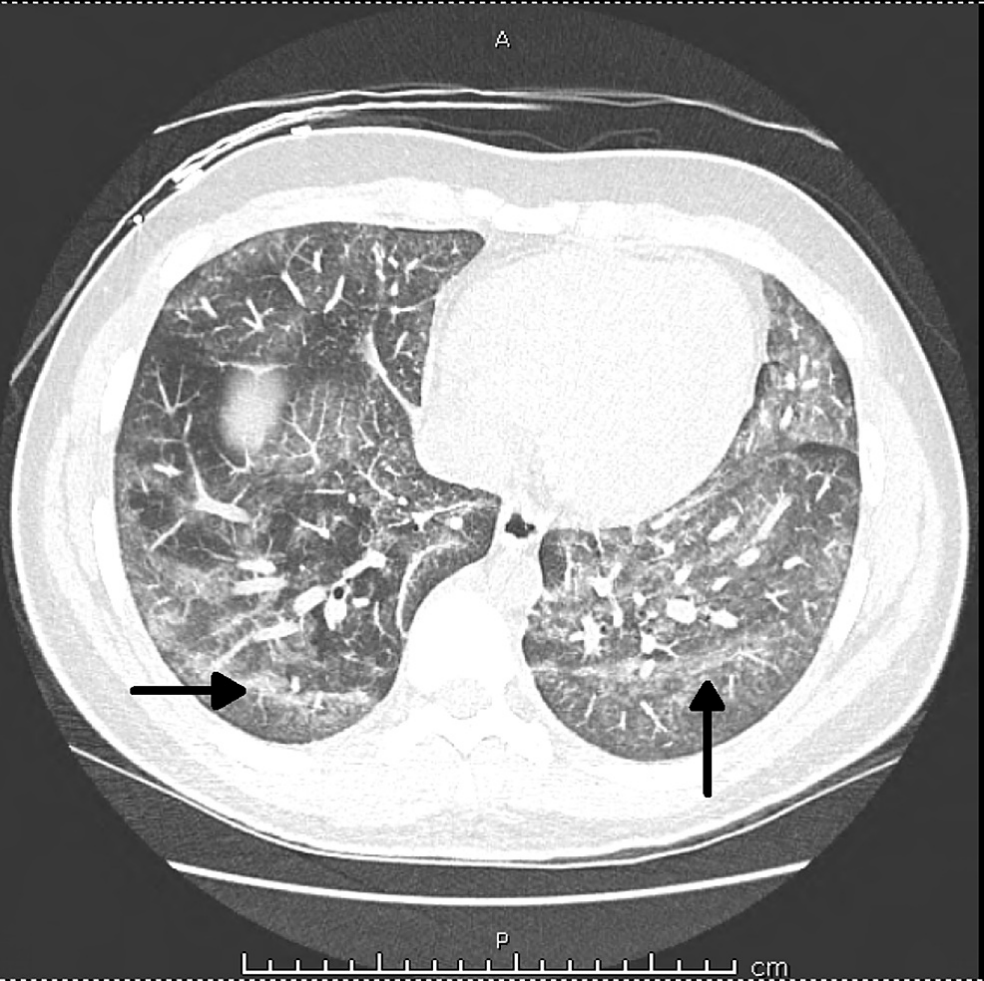
Computed tomography (CT) chest (cross-sectional view) - Bilateral lung infiltrates due to EVALI EVALI: E-cigarette or vaping product use-associated lung injury

Chest X-ray showed bilateral pneumonia (Figure 1), and computed tomography (CT) scan of the chest showed bilateral lung infiltrates (Figures 2-4).

Treatment

The patient was treated with high dose steroids (methylprednisolone), empiric antibiotics, high flow oxygen, and managed in the Intensive Care Unit (ICU) for seven days. The patient was discharged on a tapering dose of steroids (Prednisone 60 mg tapered over two weeks ) with a follow-up CT chest as an outpatient.

## Discussion

EVALI is a diagnosis of exclusion. There is no specific diagnostic test to diagnose, and it carries significant morbidity and mortality [1]. Electronic cigarettes (e-cigarettes) are battery-powered devices that allow users to inhale aerosolized liquid [4]. E-cigarettes are called vapes, e-hookahs, vape pens, tank systems, mods, and electronic nicotine delivery systems (ENDS) [2].

Vaping is the process of inhaling an aerosol that is produced by heating a liquid or wax containing substances, such as nicotine, cannabinoids (e.g., tetrahydrocannabinol, cannabidiol), flavoring, and additives (e.g., glycerol, propylene glycol) [5]. When vaping, e-liquid is exposed to high temperatures creating a suspension - aerosol; it is inaccurately called a vapor, which is the substance's gaseous state [4,6].

EVALI is an acute or subacute respiratory illness that can be severe and life-threatening [7-9] and was originally identified in the year 2019. About 80% of diagnosed individuals are under 35 years, and about 66% of patients are male [2]. Approximately 22% of patients have underlying asthma [7].

EVALI exhibits a continuum of acute lung injury with pathologic findings of acute fibrinous pneumonitis, diffuse alveolar damage, or organizing pneumonia, usually bronchiolocentric and accompanied by bronchiolitis [10]. No infectious etiology has been identified.

THC has been the widely used product (75-80%) [2,7,11,12]. Vitamin E acetate, nicotine, cannabinoid (CBD), or other plant oils are other products of use.

Respiratory symptoms include shortness of breath (85%), cough (85%), chest pain (52%), pleuritic chest pain (36%), and hemoptysis (8%) [7]. Constitutional symptoms reported are subjective fever and chills. Gastrointestinal (GI) symptoms include nausea (66%), vomiting (61%), diarrhea (44%), and abdominal pain (34%) [7].

Understanding the severity of illness, exposures like other bacterial, viral, and fungal infections, medications, travel, occupation, other comorbidities would help streamline the further workup of identified radiological opacities. Initial workup includes complete blood count with a differential and renal panel. LFT would be needed for patients presenting with GI symptoms. Excluding bacterial pneumonia and viral pneumonia is also part of the workup. Community-acquired pneumonia and acute eosinophilic pneumonia are the top differential diagnoses. It is crucial not to miss community-acquired pneumonia (CAP), as it is more common than EVALI.

Chest radiograph typically shows bilateral diffuse, hazy, or consolidative opacities [7,13]. Computed tomography angiography (CTA) chest or high-resolution CT chest will help with further characterization of the lung findings. Flexible bronchoscopy with bronchoalveolar lavage, lung biopsy help exclude other causes of progressive pneumonitis.

Empiric antibiotics and systemic corticosteroids are the mainstays of therapy [7,14,15]. A short course of systemic corticosteroid is administered with the equivalent of methylprednisolone 0.5 to 1 mg/kg per day, tapered over five to 10 days depending on the clinical course [7,14-16]. Supportive care includes supplemental oxygen with a target pulse oxygen saturation of 88 to 92 percent. Given the recurrence concern, it is advisable to monitor vitals, oxygenation, and activity tolerance at least 24-48 hours before discharge from the hospital [17]. In patients with chronic conditions, follow-up after hospital discharge optimally within 48 hours might minimize EVALI patient's risk of rehospitalization and death [17].

Fatality is reported to be high among patients over 35 years old, patients with asthma (23%), cardiac disease (47%), or a mental health condition (65%) [18]. Among patients who died, 52 percent were obese. In lieu of public health and safety, the Centers for Disease Control advise complete avoidance of tetrahydrocannabinol (THC)-containing e-cigarettes or vaping products [11].

Clinicians need to include vaping history - including vaping products, brands, and wattage settings - to ensure that potential exposures to vape products and high-risk vaping behaviors (such as the use of Dank Vape branded THC products) are identified promptly [19].

Given the diagnosis of EVALI is nonspecific, radiological detection of airway-centered acute inhalation lung injury and the detection of vitamin E acetate, and the presence of vacuolization of macrophages and pneumocytes in BAL fluid provide important diagnostic clues for this condition (Table 2) [20].

**Table 2:** Proposed criteria for EVALI EVALI: E-cigarette or vaping product use-associated lung injury; PCR: Polymerase chain reaction [3,6].

Confirmed case
Use of an e-cigarette ("vaping") or "dabbing" in the previous 90 days*
Lung opacities on chest radiograph or computed tomography
Exclusion of lung infection based on: Negative influenza PCR or rapid test (unless out of season); Negative respiratory viral panel; Negative testing for clinically indicated respiratory infections (e.g., urine antigen test for Legionella and Streptococcus pneumoniae, blood cultures, sputum cultures if producing sputum, and bronchoalveolar lavage if performed); Negative testing for HIV-related opportunistic respiratory infections (if appropriate)
Absence of a plausible alternative diagnosis (e.g., cardiac, neoplastic, rheumatologic)

## Conclusions

EVALI is a diagnosis of exclusion with no specific diagnostic test carrying significant morbidity and mortality. We emphasize the importance of being vigilant about the history of vaping in individuals presenting with acute hypoxic respiratory failure. Even though community-acquired pneumonia is more common than EVALI, having an in-depth understanding of the wide array of etiologies of bilateral lung opacities on radiological imaging, would help provide targeted therapy to the patients with favorable outcomes. Though our case presentation was from before COVID-19 pandemic, with the current pandemic COVID-19, it is easy to miss diagnosis of EVALI or as additional cause for respiratory failure. Counseling on cessation of vaping and offering them the needed support can make a difference in patients’ lives.
